# Association between rituximab treatment and outcome in nonparaneoplastic autoimmune cerebellar ataxia: an observational cohort study

**DOI:** 10.3389/fimmu.2026.1931030

**Published:** 2026-10-08

**Authors:** Mange Liu, Le Zhang, Siyuan Fan, Haitao Ren, Pinfei Ni, Yalin Ju, Lin Bai, Hongzhi Guan

**Affiliations:** Department of Neurology, Peking Union Medical College Hospital, Chinese Academy of Medical Sciences & Peking Union Medical College, Beijing, China

**Keywords:** autoimmune cerebellar ataxia, immunotherapy, prognosis, relapse, rituximab

## Abstract

**Background:**

Autoimmune cerebellar ataxia (ACA) can cause substantial disability. Evidence regarding immunotherapy for ACA, including rituximab (RTX), remains limited. This study evaluated the association of RTX treatment with outcomes in nonparaneoplastic ACA patients.

**Methods:**

ACA patients from Peking Union Medical College Hospital (April 2013–July 2025) were included if they received immunotherapy and had documented RTX exposure status and modified Rankin Scale (mRS) scores at baseline and at last follow-up. The primary outcome was ΔmRS, defined as the zenith mRS score minus the last follow-up mRS score. Secondary outcomes included favorable outcome (last follow-up mRS score of ≤ 2) and relapse. Subgroup analyses were performed in patients with and without antineural antibodies, and patients with zenith mRS scores of ≥ 4.

**Results:**

Among 144 included patients, 49 were categorized into the RTX group. Compared with the non-RTX group, the RTX group had higher zenith and last follow-up mRS scores, more often experienced relapse during the disease course, and were more likely to receive pulse corticosteroid and intravenous immunoglobulin treatment. RTX treatment was not independently associated with ΔmRS (*β* = − 0.185, 95% confidence interval [CI]: −0.721 to 0.350; *p* = 0.495) or relapse risk (HR: 0.782, 95% CI: 0.267 to 2.294; *p* = 0.654). Among patients with antineural antibodies, RTX treatment was associated with a numerically greater ΔmRS, although this association did not reach statistical significance (*β* = 0.658, 95% CI: − 0.050 to 1.365; *p* = 0.068). Relapse was consistently associated with poorer outcomes, whereas response to first-line immunotherapy was associated with better functional recovery.

**Discussion:**

In this real-world ACA cohort, we did not observe a significant association between RTX treatment and improved prognosis or reduced relapse risk, although RTX may be associated with greater mRS improvement in patients with antineural antibodies. Functional outcome analyses should be interpreted cautiously because RTX-treated patients had important baseline and disease-course imbalances suggestive of clinically more complicated and relapsing disease.

## Introduction

Autoimmune cerebellar ataxia (ACA) comprises a group of disorders in which aberrant autoimmune responses lead to cerebellar injury and represents an important cause of sporadic ataxia (16). According to the putative triggers of autoimmunity and related antineuronal antibodies, ACA can be classified into several subtypes, including paraneoplastic cerebellar degeneration (PCD), antiglutamic acid decarboxylase (GAD) antibody-associated cerebellar ataxia, and primary autoimmune cerebellar ataxia (PACA) (13).

ACA can result in substantial functional disability, imposing a considerable burden on patients and their families. Our group’s large ACA cohort study indicated that despite intensive immunotherapy, approximately half of patients remained unable to live independently, and nearly 20% experienced relapse during follow-up (11). At present, immunotherapy for ACA is largely guided by expert opinion and clinical experience. First-line immunotherapy for ACA often includes glucocorticoids, intravenous immunoglobulin (IVIg), and plasma exchange, partly based on expert consensus for autoimmune encephalitis (14, 19). However, although ACA and autoimmune encephalitis are both immune-mediated disorders of the central nervous system and may share clinical features such as subacute onset and relapse, the therapeutic analogy may not be fully transferable because the two conditions differ in their pathophysiology and antibody targets. In autoimmune encephalitis, neurological symptoms are often mediated by pathogenic neuronal surface or synaptic antibodies, whereas ACA is more heterogeneous and may involve prominent T-lymphocyte-mediated cerebellar injury (15). Therefore, whether treatment strategies derived from autoimmune encephalitis apply to ACA requires disease-specific evaluation. Rituximab (RTX) exerts marked and relatively sustained immunosuppression by targeting CD20 to deplete B lymphocytes, thereby reducing autoantibody production, and by indirectly attenuating cellular immune responses through complex immune interactions (21). Previous case reports and small case series suggest that RTX-containing regimens may ameliorate neurological dysfunction in patients with ACA (14, 20, 22). However, these reports are limited by small sample size and potential selective reporting. In a relatively larger ACA cohort, only three patients received RTX, and none showed substantial improvement (8). These limited and inconsistent data highlight the need for larger cohort studies to further clarify the potential role of RTX in ACA.

Therefore, in a large ACA cohort, we aim to evaluate whether an escalated immunotherapy regimen involving RTX can improve functional outcomes and reduce the risk of relapse, and to investigate prognostic factors for ACA. Since the prognosis of PCD patients was significantly influenced by the underlying malignancy, only nonparaneoplastic ACA patients were included. These efforts may help identify new therapeutic options and establish an individualized, high-quality treatment pathway for ACA.

## Materials and methods

### Participants

This was a real-world study based on the ACA cohort of Peking Union Medical College Hospital (11). Beginning in April 2013, patients in the Neurology Department of our institution were included if they met the diagnostic criteria for PACA, mGLuR1, or GAD antibody-related cerebellar ataxia (2, 3, 6). Patients with other neuroimmune diseases, including opsoclonus–myoclonus syndrome and autoimmune encephalitis, were included if cerebellar ataxia was the predominant or one of the major symptoms. Patients were eligible if they had (1) documented RTX exposure status during the treatment course and (2) modified Rankin Scale (mRS) assessments available at baseline (pretreatment) and at the last follow-up. Patients were excluded if they had a diagnosis of PCD, had a missing baseline or follow-up mRS score, did not receive immunotherapy, or had unavailable immunotherapy regimen information.

### Data collection

The following data were collected: age at disease onset, sex, neurological symptoms, and brain magnetic resonance imaging (MRI) findings; routine cerebrospinal fluid (CSF) examination, oligoclonal bands (OB), and CSF cytology; concomitant systemic autoimmune diseases; serum autoantibodies such as thyroglobulin antibody (anti-Tg), thyroid peroxidase antibody (anti-TPO), Sjogren’s syndrome A antibodies (anti-SSA) and Sjogren’s syndrome B antibodies (anti-SSB), and antinuclear antibody (ANA); and immunotherapy. Serum and CSF ACA antibody panels (Tr(DNER)/Zic4/ITPR1/Homer-3/neurochondrin/PKCγ/PCA-2/AP3B2/mGLuR1/ARHGAP26/CARPVIII/Rab6/EEF1D/Sez6L2 antibodies) were tested by both a cell-based assay (CBA) and a tissue-based assay (TBA) (EUROIMMUN, Lübeck, Germany), according to previously described antibody-specific assay procedures (4, 5, 12, 17). Paraneoplastic antibody assays (Hu/Yo/Ri/Ma2/Ta/CV2/amphiphysin/recoverin antibodies), autoimmune encephalitis antibodies (NMDAR/LGI1/GABAR/CASPR2/GAD65/IgLON5/DPPX/GFAP antibodies), and antibodies against aquaporin-4 and gangliosides were also examined. Serum antigluten antibody (antiendomysial and antigliadin immunoglobulin G [IgG]/IgA) testing; electromyography; and screening for infection, toxins, metabolic diseases, and hereditary ataxia were also completed if clinically appropriate to exclude other causes. mRS score was used to evaluate their neurological disability. Patients were followed regularly at the clinic or by telephone.

### Definitions of variables and outcomes

The exposure variable was RTX treatment, defined as receipt of at least one RTX infusion after baseline assessment.

The primary outcome was the difference between the zenith mRS score and the mRS score at the last follow-up (ΔmRS). A positive ΔmRS indicates improvement. The zenith mRS score was defined as the highest mRS recorded prior to initiation of immunotherapy. If the patient’s symptoms did not improve after immunotherapy, the zenith mRS score was defined as the mRS score at the time of enrollment. The last follow-up mRS was defined as the mRS recorded at the final available clinical visit.

The secondary outcomes included favorable prognosis and relapse. Since there is no widely accepted definition of relapse in ACA, we used the definition applied in autoimmune encephalitis, namely exacerbation of cerebellar ataxia after clinical improvement or stabilization for at least 2 months (19). This threshold was chosen to distinguish relapse from short-term fluctuations. Relapse events were adjudicated by two neurologists who reviewed clinical notes, mRS evolution, telephone follow-up records, and ancillary data; disagreements were resolved by consensus with a senior neurologist. For time-to-event analyses, time to relapse was calculated from symptom onset to the first relapse date; patients without relapse were censored at the last follow-up date. mRS score ≤ 2 at the last follow-up was defined as a favorable prognosis (1, 10).

Prespecified covariates included age at onset, sex, coexisting autoimmune disease (yes/no), immunotherapy regimen, zenith mRS score, follow-up duration, and overall relapse status. Response to first-line immunotherapy was defined as the response status assessed prior to RTX initiation, determined based on a combination of patient-reported change, physician observation, and medical record documentation.

### Statistical analysis

Baseline characteristics were summarized using median (IQR) for continuous variables due to their nonnormal distribution and counts (percentages) for categorical variables. The Mann–Whitney *U* test was used for continuous variables. The Pearson *χ*^2^ test or Fisher’s exact test was used for categorical variables, as appropriate.

For the primary analysis of the association between RTX and ΔmRS, we used a multivariable linear regression model with ΔmRS as the dependent variable and RTX use as the main independent variable. The fully adjusted model included age at onset, sex, coexisting autoimmune diseases, zenith mRS score, immunotherapy regimen, effectiveness of first-line immunotherapy, follow-up duration, and overall relapse status during the disease course. Overall relapse status was included to account for a relapsing disease course and treatment-escalation background; because RTX was sometimes initiated after relapse, this variable should not be interpreted as a simple post-RTX outcome. Tolerance and variance inflation factor values were calculated to evaluate potential multicollinearity among treatment-related covariates.

To address potential confounding by indication, a propensity-score-matched sensitivity analysis was performed for RTX treatment. Propensity scores were estimated using binary logistic regression with sex, age at onset, and zenith mRS score as covariates. One-to-one matching was performed with a caliper of 0.020. Covariate balance before and after matching was assessed using standardized mean differences. Multivariable linear regression was repeated in the propensity-score-matched cohort. In addition, because response to first-line immunotherapy and overall relapse could be conceptually related to functional outcome or could act as mediating variables, a revised multivariable linear regression model excluding these two variables was performed as a sensitivity analysis.

For the relapse analysis, RTX exposure was modeled as a time-dependent covariate in the Cox proportional hazards model. Time zero was defined as symptom onset. For patients who received RTX, follow-up time before the first RTX infusion was coded as unexposed, and follow-up time after the first RTX infusion was coded as RTX-exposed. Patients who experienced first relapse before RTX initiation contributed an event during unexposed person-time and did not contribute post-RTX risk time in the first-relapse analysis. Patients without relapse were censored at the last follow-up. The Cox model was adjusted for age at onset, sex, zenith mRS score, and effectiveness of first-line immunotherapy. A sensitivity Cox model excluding effectiveness of first-line immunotherapy was also performed. Binary logistic regression was used to identify factors associated with favorable outcome. Given that only 51 patients had an unfavorable outcome, a parsimonious model including four *a priori* selected covariates: age at onset, sex, zenith mRS score, and RTX treatment was applied. Results are presented as odds ratios with 95% confidence intervals.

In addition, to assess the association between RTX and functional outcome in ACA patients with severe disease, we performed a subgroup analysis restricted to patients with a zenith mRS score of ≥ 4. The same fully adjusted linear regression model was applied in this subgroup analysis.

Exploratory subgroup and interaction analyses were performed to evaluate whether the association between RTX treatment and functional improvement differed according to ACA immunological status. Fully adjusted multivariable linear regression models were fitted separately in patients with and without antineural antibody positivity (defined as patients who either had a specifically identified antineural antibody or showed positive neural tissue reactivity on TBA despite negative CBA findings) using the same covariates as in the primary adjusted model. An interaction term between RTX treatment and antineural antibody status was added to the fully adjusted model in the overall cohort.

Among patients who received RTX, we further performed an exploratory responder analysis. RTX response was determined retrospectively from medical-record documentation of clinical improvement after RTX initiation, supported by patient-reported change, physician assessment, and mRS evolution. Clinical characteristics were compared between RTX responders and nonresponders.

Statistical analyses were performed using SPSS version 26.0. A two-sided *p*-value < 0.05 was considered statistically significant. No imputation was performed for missing covariate data. Regression analyses were conducted using complete-case analysis.

## Results

From April 2013 to July 2025, 266 ACA patients were enrolled in our cohort. Patients were excluded for an unavailable zenith mRS score (*n* = 23), an unavailable last follow-up mRS score due to loss to follow-up (*n* = 39), missing immunotherapy regimen information (*n* = 4), diagnosis of PCD (*n* = 48), or not receiving immunotherapy (*n* = 8). Therefore, 144 patients with ACA were included in the analysis.

Fifty-nine (41.0%) patients were men. The median age at onset was 39.5 (IQR: 24.3–50.0) years. Extracerebellar involvement included pyramidal signs in 75, diplopia/ophthalmoplegia in 51, and peripheral neuropathy/radiculopathy in 19 patients. Sixty-one patients experienced prodromal infection-like symptoms. Malignancy was detected in two patients (both thyroid cancer), and 31 patients had coexisting autoimmune diseases, including Sjogren syndrome in 11, Hashimoto’s thyroiditis in 11, and other autoimmune diseases in nine. The distribution of ACA subtypes was as follows: PACA in 105 (72.9%), opsoclonus–myoclonus syndrome in four (2.8%), autoimmune encephalitis-related cerebellar ataxia in 13 (9.0%), anti-GAD antibody-associated cerebellar ataxia in 10 (6.9%), and anti-mGLuR1 antibody-associated cerebellar ataxia in 12 (8.3%), as shown in **Figure 1**. Known antineural antibodies were detected in serum and/or CSF in 62 (43.1%, 20 in the RTX group and 42 in the non-RTX group) patients, and another 19 patients (seven in the RTX group and 12 in the non-RTX group) were positive on TBA but negative on CBA (**Supplementary Table S1**). The median CSF white blood cell count and protein concentration were 6 (IQR: 2–30)/μL and 0.50 (IQR: 0.35–0.79) g/L, respectively. CSF-specific oligoclonal bands were present in 80 (55.6%) patients. Brain MRI was unremarkable in 71 (50.4%) patients, while cerebellar atrophy and abnormal signals were observed in 26 (18.4%) and 44 (31.2%) patients, respectively.

**Figure 1:**
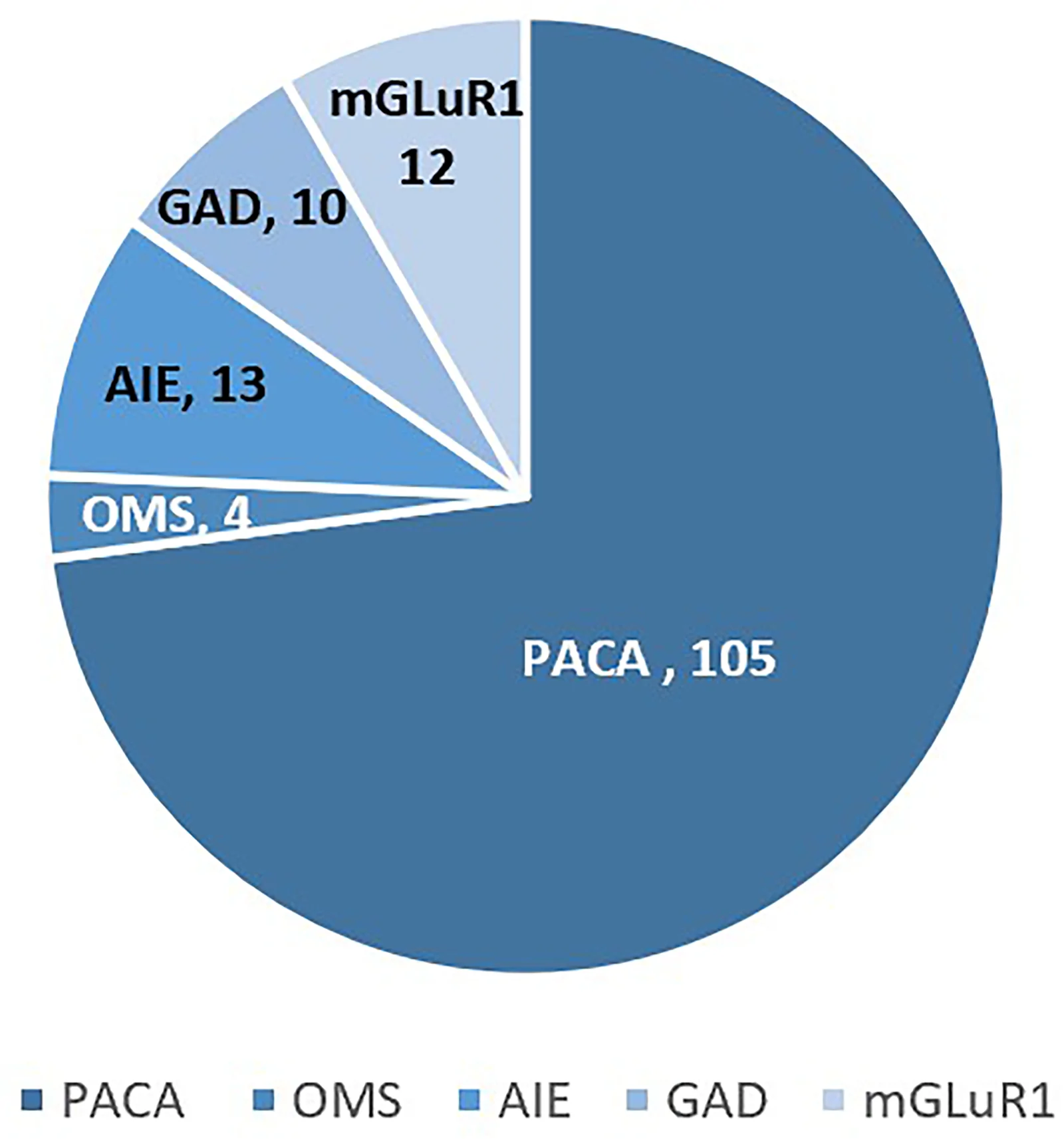
The distribution of autoimmune cerebellar ataxia subtypes. AIE, autoimmune encephalitis-related cerebellar ataxia; GAD, antiglutamic acid decarboxylase antibody-related cerebellar ataxia; mGLuR1, anti-mGLuR1-related cerebellar ataxia; OMS, opsoclonus–myoclonus syndrome; PACA, primary autoimmune cerebellar ataxia.

In terms of immunotherapy, 136 patients received corticosteroids and 114 received IVIg. RTX and mycophenolate mofetil (MMF) were prescribed to 49 and 74 patients as long-term immunotherapy, respectively. Various immunosuppressants, including cyclophosphamide, tacrolimus, and efgartigimod, were used in another three patients. The median interval from symptom onset to the first RTX infusion was 10 months (IQR: 4–21.5 months). On average, each patient received 1.41 RTX infusions, and the mean dose per infusion was 560 mg. The median follow-up duration from ataxia symptom onset was 26.5 months (IQR: 14.3–45.8 months). The median zenith and last follow-up mRS scores were 4 (IQR: 2–4) and 2 (IQR: 1–4), respectively. Overall, 93 patients achieved a favorable prognosis, and 45 patients relapsed during the overall disease course. Among the 49 RTX-treated patients, 21 experienced relapse before the first RTX infusion and four experienced relapse after RTX initiation; 24 RTX-treated patients had no relapse.

Baseline characteristics stratified by RTX treatment are summarized in **Table 1**. Compared with the patients in the non-RTX group, those in the RTX group had higher zenith mRS scores (4 [3.5–4.5] vs. 4 (2–4); *p* = 0.005) and higher last follow-up mRS scores (2 [2–4] vs. 2 [1–4]; *p* = 0.001). Patients in the RTX group more frequently received pulse corticosteroids (91.8% vs. 61.1%; *p* < 0.001) and IVIg (91.8% vs. 72.6%; *p* = 0.007). Relapse during the overall disease course was more common in the RTX group than in the non-RTX group (51% vs. 21.1%; *p* < 0.001), whereas sex, age at onset, coexisting autoimmune disease, MMF use, response to first-line immunotherapy, and follow-up duration did not differ significantly between groups.

**Table 1:** Baseline characteristics of patients according to rituximab treatment.

Characteristic	RTX group (*n* = 49)	Non-RTX group (*n* = 95)	*p*-value
Male (no.; %)	21 (42.9)	38 (40.0)	0.741
Age at onset (years)	35 (23, 45)	42 (28, 52)	0.103
Coexisting autoimmune diseases (no.; %)	8 (16.3)	23 (24.5)	0.262
Antineural antibody-positive^a^ (no.; %)	27 (55.1)	54 (56.8)	0.842
Identified antineural antibody	20 (40.8)	42 (44.2)	
TBA-positive but CBA-negative	7 (14.3)	12 (12.6)	
Zenith mRS score	4 (3.5, 4.5)	4 (2, 4)	**0.005**
Last follow-up mRS score	2 (2, 4)	2 (1, 4)	**0.001**
Immunotherapy (no.; %)			
Pulse corticosteroid	45 (91.8)	58 (61.1)	**< 0.001**
Nonpulse corticosteroid^b^	12 (24.5)	39 (41.1)	**0.049**
IVIg	45 (91.8)	69 (72.6)	**0.007**
MMF	27 (55.1)	47 (49.5)	0.522
Effectiveness of first-line immunotherapy (no.; %)	41 (83.7)	82 (86.3)	0.67
Follow-up duration (months)	26 (15.5, 37)	27 (12, 54)	0.856
Relapse (no.; %)	25 (51.0)	20 (21.1)	**< 0.001**

*ACA*, autoimmune cerebellar ataxia; *IVIg*, intravenous immunoglobulin; *MMF*, mycophenolate mofetil; *mRS*, modified Rankin Scale; *RTX*, rituximab.

a For subgroup analyses, the antineural antibody-positive subgroup was operationally defined as patients who either had specifically identified antineural antibodies or showed positive neural tissue reactivity on TBA despite negative CBA findings.

b Nonpulse corticosteroid therapy refers to acute-phase immunotherapy with methylprednisolone at a dose of < 500 mg/day, or an equivalent corticosteroid dose, during the initial attack or relapse.

The bold values indicate statistical significance (p < 0.05).

### Primary outcome

In the fully adjusted multivariable linear regression model, several factors were associated with ΔmRS, defined as the difference between zenith mRS and last follow-up mRS (**Table 2**). RTX treatment was not independently associated with ΔmRS (*β* = − 0.185, 95% confidence interval [CI]: − 0.721 to 0.350; *p* = 0.495).

**Table 2:** Multivariable linear regression analysis of factors associated with ΔmRS with collinearity diagnostics.

Variables	Regression coefficient (*β*)	95% CI	*p*-value	Tolerance	VIF
Age at onset (year)	− 0.004	− 0.019 to 0.012	0.631	0.733	1.365
Male vs. female	0.613	0.136 to 1.089	**0.012**	0.868	1.152
Coexisting autoimmune diseases	0.074	− 0.502 to 0.649	0.800	0.844	1.185
Zenith mRS score	0.485	0.275 to 0.695	**< 0.001**	0.866	1.155
Immunotherapy					
RTX	− 0.185	− 0.721 to 0.350	0.495	0.735	1.361
Pulse corticosteroid	− 0.500	− 1.168 to 0.169	0.141	0.527	1.896
Nonpulse corticosteroid	− 0.140	− 0.749 to 0.470	0.651	0.562	1.778
IVIg	0.068	− 0.510 to 0.646	0.815	0.857	1.167
MMF	− 0.301	− 0.771 to 0.169	0.208	0.859	1.164
Effectiveness of first-line immunotherapy	1.378	0.708 to 2.047	**< 0.001**	0.845	1.183
Follow-up duration (month)	− 0.008	− 0.017 to 0.001	0.086	0.810	1.235
Relapse	− 0.793	− 1.382 to − 0.205	**0.009**	0.643	1.555

*ΔmRS* = zenith mRS score − last follow-up mRS score; positive *β* indicates greater improvement. *R*^2^ = 0.365; adjusted *R*^2^ = 0.307.

*CI*, confidence interval; *IVIg*, intravenous immunoglobulin; *MMF*, mycophenolate mofetil; *mRS*, modified Rankin Scale; *RTX*, rituximab; *VIF*, variance inflation factor.

The bold values indicate statistical significance (p < 0.05).

Male sex was associated with greater mRS improvement (*β* = 0.613, 95% CI: 0.136 to 1.089; *p* = 0.012), whereas overall relapse during the disease course was independently associated with smaller mRS improvement (*β* = − 0.793, 95% CI: − 1.382 to − 0.205; *p* = 0.009). A higher zenith mRS score was associated with greater ΔmRS (*β* = 0.485, 95% CI: 0.275 to 0.695; *p* < 0.001), and the effectiveness of first-line immunotherapy was also associated with greater improvement (*β* = 1.378, 95% CI: 0.708 to 2.047; *p* < 0.001). Collinearity diagnostics did not indicate substantial multicollinearity among the treatment variables; the highest VIF was 1.896 for pulse corticosteroid treatment and the lowest tolerance value was 0.527.

In the revised multivariable linear regression model excluding response to first-line immunotherapy and overall relapse, RTX treatment remained not significantly associated with ΔmRS (*β* = − 0.499, 95% CI: − 1.037 to 0.040; *p* = 0.069, **Supplementary Table S2**). Follow-up duration was statistically associated with ΔmRS (*β* = − 0.012 per month, 95% CI: − 0.021 to − 0.003; *p* = 0.011).

After 1:1 propensity-score matching, 43 matched pairs, including 86 patients, were retained. Covariate balance improved after matching, with absolute standardized mean differences below 0.10 for sex, age at onset, and zenith mRS score (**Supplementary Table S3**). In the propensity-score-matched cohort, RTX treatment was not significantly associated with ΔmRS (*β* = − 0.200, 95% CI: − 0.988 to 0.588; *p* = 0.614; **Supplementary Table S4**).

### Secondary outcomes

During follow-up, 93 patients achieved a favorable prognosis, defined as a last follow-up mRS score ≤ 2, and 45 patients experienced relapse during the overall disease course.

In the time-dependent Cox regression analysis evaluating relapse in relation to RTX exposure, RTX exposure was not significantly associated with relapse risk (hazard ratio [HR]: 0.782, 95% CI: 0.267 to 2.294; *p* = 0.654; **Table 3**). Unadjusted Kaplan–Meier curves according to RTX treatment status are shown in **Figure 2**, with no significant difference between groups (log-rank *p* = 0.363). Older age at onset was associated with a lower risk of relapse (HR: 0.965, 95% CI: 0.947 to 0.984; *p* < 0.001). Sex, zenith mRS score, and response to first-line immunotherapy were not significantly associated with relapse risk. In a sensitivity Cox model excluding response to first-line immunotherapy, the association between time-dependent RTX exposure and relapse remained nonsignificant.

**Table 3:** Cox regression analysis of predictors of relapse in the time-to-event analysis.

Variables	HR (95% CI)	*p*-value
RTX exposure as a time-dependent covariate	0.782 (0.267 to 2.294)	0.654
Age at onset/year	0.965 (0.947 to 0.984)	**< 0.001**
Male vs. female	1.021 (0.558 to 1.867)	0.947
Zenith mRS score	1.014 (0.770 to 1.335)	0.923
Effectiveness of first-line immunotherapy	6.729 (0.905 to 50.041)	0.063

For RTX-exposed patients, only relapses occurring after RTX initiation were attributed to RTX exposure; relapses before RTX initiation were not counted as post-RTX events. Concordance index = 0.713.

*CI*, confidence interval; *HR*, hazard ratio; *mRS*, modified Rankin Scale; *RTX*, rituximab.

The bold values indicate statistical significance (p < 0.05).

**Figure 2:**
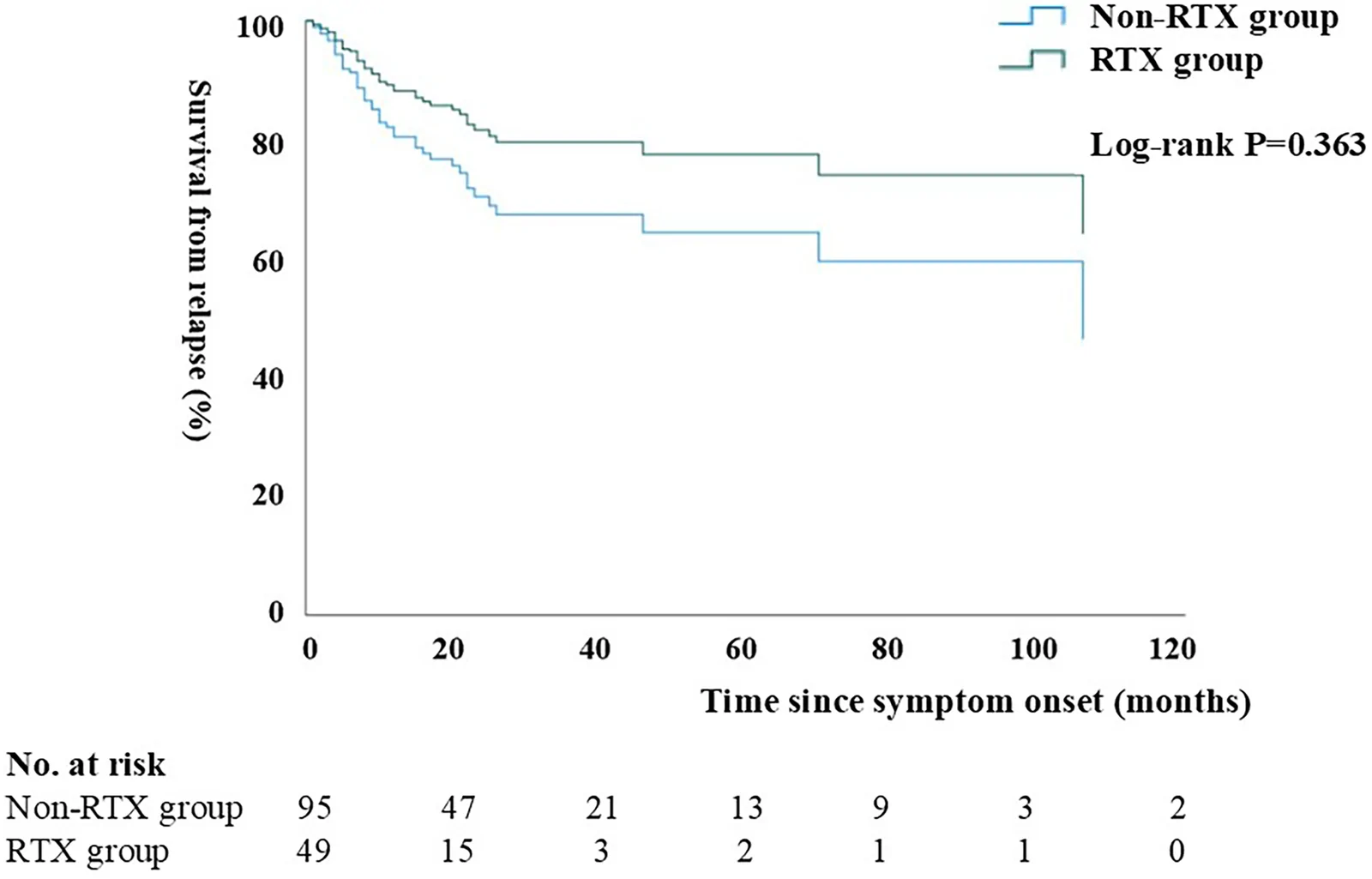
Kaplan–Meier curves showing relapse-free survival in patients grouped according to rituximab (RTX) treatment. Differences between groups were assessed using the log-rank test.

In the binary logistic regression model for favorable outcome, a higher zenith mRS score was independently associated with lower odds of achieving a favorable outcome at the last follow-up (odds ratio [OR]: 0.490, 95% CI: 0.335 to 0.716; *p* < 0.001; **Table 4**). RTX treatment was not significantly associated with favorable outcome (OR: 0.615; 95% CI: 0.281 to 1.348; *p* = 0.225).

**Table 4:** Binary logistic regression analysis of factors associated with favorable outcome.

Variables	OR	95% CI	*p*-value
Age at onset (year)	0.996	0.974 to 1.019	0.756
Male vs. female	1.613	0.753 to 3.457	0.219
Zenith mRS score	0.490	0.335 to 0.716	**< 0.001**
RTX treatment	0.615	0.281 to 1.348	0.225

Favorable outcome was defined as an mRS score (≤ 2) at the last follow-up. OR values > 1 indicate higher odds of favorable outcome. − 2 log-likelihood = 165.55; Nagelkerke *R*^2^ = 0.192.

*CI*, confidence interval; *mRS*, modified Rankin Scale; *OR*, odds ratio; *RTX*, rituximab.

The bold values indicate statistical significance (p < 0.05).

### Subgroup analyses

Among patients with severe disease, defined as zenith mRS score ≥ 4, 88 patients were included in the subgroup analysis (**Supplementary Table S6**). RTX treatment was not independently associated with ΔmRS (*β* = − 0.314, 95% CI, − 0.943 to 0.314; *p* = 0.323). Male sex and effectiveness of first-line immunotherapy were associated with greater improvement, whereas MMF treatment and overall relapse were associated with smaller ΔmRS.

The association between RTX treatment and ΔmRS differed according to antineural antibody status. Among patients classified as antineural antibody-positive according to the above operational definition (*n* = 81), RTX treatment was associated with a numerically greater ΔmRS, although this association did not reach conventional statistical significance (*β* = 0.658, 95% CI: − 0.050 to 1.365; *p* = 0.068; **Supplementary Table S7**). In contrast, among patients without antineural antibodies (*n* = 63), RTX treatment was associated with a significantly lower ΔmRS (*β* = − 1.329, 95% CI: − 2.194 to − 0.464; *p* = 0.003; **Supplementary Table S8**). When the RTX × serostatus interaction term was added to the fully adjusted model in the overall cohort, the interaction term was statistically significant (*β* = 1.797, 95% CI: 0.874 to 2.719; *p* < 0.001; **Supplementary Table S9**), indicating that the association of RTX with functional outcome was significantly more favorable in patients with antineural antibodies than in those without antineural antibodies.

### Exploratory RTX responder analysis

Among 49 RTX-treated patients, 32 were classified as RTX responders and 17 as nonresponders (**Table 5**). Most baseline and pretreatment characteristics were similar between groups, including sex, age at onset, coexisting autoimmune diseases, ACA subtype, neurological features, MRI findings, CSF profile, use of concomitant immunotherapies, effectiveness of first-line immunotherapy, and follow-up duration. As expected, responders had a lower last follow-up mRS score than nonresponders (2 [IQR: 2–4] vs. 4 [IQR: 2–4]; *p* = 0.032), with a borderline difference in ΔmRS (1 [IQR: 0–2] vs. 0 [IQR: 0–1.5]; *p* = 0.081). Serum immunoglobulin G (IgG) concentration, neutrophil and lymphocyte counts before RTX treatment were also comparable between responders and nonresponders (**Supplementary Table S10**).

**Table 5:** Clinical characteristics of RTX responders and nonresponders.

Characteristic	RTX responders (*n* = 32)	RTX nonresponders (*n* = 17)	*p*-value
Male (no.; %)	14 (43.8)	7 (41.2)	0.862
Age at onset (years)	33.5 (23, 46.8)	40 (21, 44)	0.916
Coexisting autoimmune diseases (no.; %)	5 (15.6)	3 (17.6)	> 0.999
ACA subtype (no.; %)			
PACA	24 (75.0)	16 (94.1)	0.464
AIE-CA	4 (12.5)	1 (5.9)	
GAD-CA	1 (3.1)	0 (0)	
mGLuR1-CA	3 (9.4)	0 (0)	
Pyramidal signs (no.; %)	25 (78.1)	13 (76.5)	> 0.999
Peripheral neuropathy/radiculopathy (no.; %)	6 (18.8)	3 (17.6)	> 0.999
Prodromal infection-like symptoms (no.; %)	19 (59.4)	9 (52.9)	0.665
Zenith mRS score	4 (3, 4.8)	4 (4, 4.5)	0.723
Last follow-up mRS score	2 (2, 4)	4 (2, 4)	**0.032**
ΔmRS	1 (0, 2)	0 (0, 1.5)	0.081
Brain MRI (no.; %)			
Unremarkable	14 (45.2)	9 (52.9)	0.910
Atrophy	4 (12.9)	1 (5.9)	
Abnormal signal	13 (41.9)	7 (41.2)	
CSF			
White blood cell count (/µL)	23.5 (6.3, 77.3)	24 (3.5, 81.5)	0.992
Protein concentration (g/L)	0.77 (0.51, 1.30)	0.56 (0.35, 1.24)	0.224
CSF-specific OB (no.; %)	23 (79.3)	12 (75.0)	> 0.999
Immunotherapy (no.; %)			
Pulse corticosteroid	30 (93.8)	15 (88.2)	0.902
Nonpulse corticosteroid	9 (28.1)	3 (17.6)	0.643
IVIg	28 (87.5)	17 (100)	0.331
MMF	16 (50.0)	11 (64.7)	0.325
Effectiveness of first-line immunotherapy (no.; %)	25 (78.1)	16 (94.1)	0.300
Follow-up duration (months)	24.5 (15.3, 37)	30 (16, 46.5)	0.667
Relapse (no.; %)	14 (43.8)	11 (64.7)	0.162

ΔmRS = zenith mRS score − last follow-up mRS score.

*ACA*, autoimmune cerebellar ataxia; *AIE-CA*, autoimmune encephalitis-related cerebellar ataxia; *CSF*, cerebrospinal fluid; *GAD-CA*, antiglutamic acid decarboxylase antibody-related cerebellar ataxia; *IVIg*, intravenous immunoglobulin; *mGLuR1-CA*, anti-mGLuR1-related cerebellar ataxia; *MMF*, mycophenolate mofetil; *mRS*, modified Rankin Scale; *MRI*, magnetic resonance imaging; *OB*, oligoclonal bands; *OMS*, opsoclonus–myoclonus syndrome; *PACA*, primary autoimmune cerebellar ataxia; *RTX*, rituximab.

### Safety of RTX treatment

Among the 49 RTX-treated patients, adverse events recorded during RTX treatment included infusion reactions in four patients, mainly fever and rash. Infection during RTX treatment was recorded in two patients, including urinary tract infection in one patient and pneumonia in another. As adverse events were identified retrospectively from medical records and spontaneous patient reports rather than through systematic safety surveillance, these findings were descriptive.

## Discussion

In this real-world cohort of patients with autoimmune cerebellar ataxia, neither the fully adjusted functional outcome analysis nor the revised time-to-event analysis demonstrated an independent favorable association between RTX and clinical outcomes. After modeling RTX exposure as a time-dependent covariate, RTX was not significantly associated with relapse risk (HR: 0.782, 95% CI: 0.267–2.294; *p* = 0.654). Although the point estimate was below 1.0, the wide confidence interval included both a potentially meaningful reduction and an increased relapse hazard, indicating substantial statistical uncertainty. Therefore, this finding should be interpreted as hypothesis-generating rather than evidence of a relapse-preventive effect.

One possible explanation is that RTX may have a true but modest biological effect in reducing relapse that our cohort was underpowered to detect, particularly given the limited number of relapse events, variable timing of RTX initiation, and heterogeneity in background immunotherapy. Alternatively, the apparent direction of effect may represent a spurious trend attributable to residual confounding. Since relapse was consistently associated with poorer outcomes in our regression models, relapse prevention remains clinically important. On the basis of the HR observed in the present study, several hundred relapse events would be required to adequately evaluate whether RTX significantly reduces relapse risk, highlighting the need for a substantially larger prospective multicenter cohort, ideally with standardized treatment timing and systematic adjustment for baseline severity and concomitant therapies, to determine whether RTX has a true relapse-reducing effect in ACA.

A key issue in interpreting the present findings is confounding by indication. Patients in the RTX group had higher zenith and last follow-up mRS scores, were more likely to relapse during the disease course, and more often received pulse corticosteroids and IVIg. These features suggest that RTX was used as escalation therapy in patients with more severe, relapsing, or clinically complicated disease rather than being randomly assigned. In addition, because RTX treatment was analyzed as an ever/never variable in the logistic regression model, time-related bias, including potential immortal time bias, cannot be excluded. Specifically, the model did not fully account for the timing of the treatment relative to disease onset, relapse, clinical worsening, and outcome assessment. Therefore, the poorer apparent functional outcomes among RTX-treated patients may be partly related to a relapsing disease course and treatment-escalation background. In **Table 2** and **Supplementary Tables S4, 6–9**, relapse referred to relapse during the overall disease course, and in many patients RTX was initiated after relapse. Therefore, relapse in these models should be considered a marker of relapsing disease course and treatment escalation background, rather than a simple post-treatment outcome.

Relapse was also consistently associated with poorer functional outcome. In the full cohort and subgroup analyses, overall relapse during the disease course was associated with smaller ΔmRS. These findings suggest that a relapsing disease course may contribute to cumulative neurological disability and nonrestorable cerebellar function in ACA (16). They also support the clinical importance of early identification of patients at high risk of relapse and careful long-term monitoring.

Response to first-line immunotherapy was strongly associated with better long-term functional outcome, similar to the findings of our PACA cohort (10). Across the main and subgroup analyses, the effectiveness of first-line immunotherapy was associated with greater ΔmRS. This suggests that early immunotherapy responsiveness may serve as a clinically useful prognostic marker in ACA. Patients who respond to first-line treatment may have a more reversible inflammatory component, whereas nonresponders may have more advanced neuronal injury or a less immunotherapy-responsive disease mechanism.

The present exploratory subgroup and interaction analyses suggest that the association between RTX treatment and functional improvement may differ according to antineural antibody status. RTX was associated with greater functional improvement in patients with antineural antibodies than in those without antineural antibodies, and the RTX × serostatus interaction was statistically significant. Some ACA-associated antibodies are thought to be more directly involved in disease mechanisms. For example, mGluR1 antibodies have experimental evidence supporting a direct pathogenic role in cerebellar motor dysfunction (18). For Sez6L2 antibodies and GAD65 antibodies, mechanistic studies suggest possible pathogenic relevance, but the results are still controversial (2, 9). However, many other antibodies detected in autoimmune cerebellar ataxia, particularly antibodies against intracellular antigens or poorly characterized antibodies, may serve primarily as biomarkers of immune-mediated disease rather than direct effectors of neuronal injury (6). Thus, the effectiveness of RTX in anti-neural antibody-positive ACA patients cannot simply be attributed to B-cell depletion and subsequent reduction in antibody titers, but may instead reflect more complex differences in immune mechanisms and treatment responsiveness across ACA subtypes. Moreover, in our cohort, antibody-positive and antibody-negative patients did not differ significantly in sex, age at onset, interval from onset to RTX treatment, zenith and last follow-up mRS scores, ΔmRS, relapse, or response to first-line immunotherapy, although MMF use was more frequent in antibody-positive patients (data not shown). Therefore, the observed interaction might not be readily explained by differences in baseline severity. Nevertheless, residual confounding by treatment selection, treatment sequence, and unmeasured disease biology cannot be excluded.

Several treatment-related findings should be interpreted with caution. MMF treatment was associated with poorer outcomes in some models. Similar to RTX, this association may reflect treatment selection in clinical practice, because patients with more severe, refractory, or relapsing disease are more likely to receive long-term immunosuppression. Therefore, these results should not be interpreted as evidence that MMF is harmful. Instead, previous studies have suggested the potential effectiveness of MMF in PACA patients (7). These findings emphasize the difficulty of estimating treatment effects in retrospective or real-world observational cohorts without randomization. Follow-up duration was also significantly related to ΔmRS in some models, but the magnitude was very small, corresponding to approximately 0.2 mRS points per year, and was therefore considered unlikely to be clinically meaningful.

This study has several limitations. First, this was a real-world observational study, and treatment allocation was not randomized. Residual confounding and confounding by indication are therefore possible, as reflected by the baseline imbalances between RTX-treated and non-RTX-treated patients. Second, the timing, dose, and duration of RTX treatment were not uniform, and some patients received RTX after relapse or after failure of other immunotherapies. Third, RTX was frequently combined with IVIg, pulse corticosteroid therapy, or corticosteroid dose adjustment, making it difficult to isolate RTX-specific effects, particularly in the exploratory responder analysis. Fourth, mRS is not an ataxia-specific scale and may not fully capture cerebellar-specific impairment. Moreover, as an ordinal rather than interval scale with limited granularity, identical one-point changes at different positions on the mRS cannot necessarily be assumed to represent equivalent clinical improvement. Future studies with ataxia-specific scale assessments, such as the Scale for the Assessment and Rating of Ataxia (SARA), at multiple time points during the disease course, may assess immunotherapy response and relapse more precisely and reduce the influence of subjective factors. In addition, this was a single-center study from a tertiary referral hospital, which may limit generalizability. Moreover, 39 patients were excluded because of missing follow-up mRS scores, and potential selection bias related to loss to follow-up cannot be excluded. The Cox regression analysis for relapse was also limited by the relatively small number of relapse events, resulting in limited statistical power and wide confidence intervals.

## Conclusion

In this observational cohort study of ACA patients, RTX was not associated with significant improvement in prognosis or a reduced risk of relapse, although it may be associated with greater improvement in mRS among patients with antineural antibodies. Functional outcome associations with RTX were difficult to interpret because RTX-treated patients showed more severe disability, more frequent relapse, and more intensive concomitant immunotherapy. Relapse was consistently associated with unfavorable outcomes, whereas response to first-line immunotherapy was associated with better functional recovery. Prospective studies with standardized treatment regimens are needed to clarify the role of RTX in ACA and to identify patients most likely to benefit from escalated immunotherapy.

## Data Availability

The raw data supporting the conclusions of this article will be made available by the authors, without undue reservation.

## References

[1] Damato V Papi C Spagni G Evoli A Silvestri G Masi G . Clinical features and outcome of patients with autoimmune cerebellar ataxia evaluated with the Scale for the Assessment and Rating of Ataxia. Eur J Neurol. (2021) 29(2):564–572. doi: 10.1111/ene.15161 34710286 PMC9564532

[2] Graus F Saiz A Dalmau J . GAD antibodies in neurological disorders - insights and challenges. Nat Rev Neurol. (2020) 16:353–65. doi: 10.1038/s41582-020-0359-x 32457440

[3] Graus F Vogrig A Muñiz-Castrillo S Antoine J-C Desestret V Dubey D . Updated diagnostic criteria for paraneoplastic neurologic syndromes. Neurol Neuroimmunol Neuroinflamm. (2021) 8. doi: 10.1212/nxi.0000000000001014 34006622 PMC8237398

[4] Guo L Ren H Fan S Chao X Liu M Guan H . Autoantibodies against eukaryotic translation elongation factor 1 delta in two patients with autoimmune cerebellar ataxia. Front Immunol. (2023) 14:1289175. doi: 10.1101/2023.09.05.556333 38332912 PMC10850295

[5] Guo L Ren H Fan S Guan H Wang J . Autoantibody against the Rab6A/Rab6B in primary autoimmune cerebellar ataxia associated with Sjogren's syndrome: A case report. J Neuroimmunol. (2021) 359:577667. doi: 10.1016/j.jneuroim.2021.577667 34332492

[6] Hadjivassiliou M Graus F Honnorat J Jarius S Titulaer M Manto M . Diagnostic criteria for primary autoimmune cerebellar ataxia-guidelines from an International Task Force on immune-mediated cerebellar ataxias. Cerebellum. (2020) 19:605–10. doi: 10.1007/s12311-020-01132-8 32328884 PMC7351847

[7] Hadjivassiliou M Grunewald RA Shanmugarajah PD Sarrigiannis PG Zis P Skarlatou V . Treatment of primary autoimmune cerebellar ataxia with mycophenolate. Cerebellum. (2020) 19(5):680–684. doi: 10.1007/s12311-020-01152-4 32524518 PMC7471147

[8] Jones AL Flanagan EP Pittock SJ Mandrekar JN Eggers SD Ahlskog JE . Responses to and outcomes of treatment of autoimmune cerebellar ataxia in adults. JAMA Neurol. (2015) 72:1304–12. doi: 10.1001/jamaneurol.2015.2378 26414229

[9] Landa J Guasp M Petit-Pedrol M Martínez-Hernández E Planagumà J Saiz A . Seizure-related 6 homolog like 2 autoimmunity: Neurologic syndrome and antibody effects. Neurol Neuroimmunol Neuroinflamm. (2021) 8. doi: 10.1212/NXI.0000000000000916 PMC7641326 33144342

[10] Liu M Ren H Wang L Fan S Bai L Guan H . Prognostic and relapsing factors of primary autoimmune cerebellar ataxia: a prospective cohort study. J Neurol. (2023) 271(3):1072–1079. doi: 10.1007/s00415-023-12128-9 38141127

[11] Liu M Ren H Zhu Y Fan S Bai L Wang J . Autoimmune cerebellar ataxia: Etiology and clinical characteristics of a case series from China. Cerebellum. (2022) 22(3):379–385. doi: 10.1007/s12311-022-01412-5 35618871

[12] Mange L Haitao R Lixin Z Siyuan F Jing W Hongzhi G . Cerebellar ataxia and myeloradiculopathy associated with AP3B2 antibody: a case report and literature review. J Neurol. (2021) 268(11):4163–4169. doi: 10.1007/s00415-021-10496-8 33988764

[13] Manto M Mitoma H . Recent advances in immune-mediated cerebellar ataxias: Pathogenesis, diagnostic approaches, therapies, and future challenges-editorial. Brain Sci. (2023) 13. doi: 10.3390/brainsci13121626 38137074 PMC10741786

[14] Mehdiyeva A Hietaharju A Sipilä J . SEZ6L2 antibody-associated cerebellar ataxia responsive to sequential immunotherapy. Neurol Neuroimmunol Neuroinflamm. (2022) 9. doi: 10.1212/nxi.0000000000001131 35031588 PMC8759717

[15] Mitoma H Adhikari K Aeschlimann D Chattopadhyay P Hadjivassiliou M Hampe CS . Consensus paper: Neuroimmune mechanisms of cerebellar ataxias. Cerebellum. (2016) 15:213–32. doi: 10.1007/s12311-015-0664-x PMC4591117 25823827

[16] Mitoma H Manto M Hampe CS . Immune-mediated cerebellar ataxias: Practical guidelines and therapeutic challenges. Curr Neuropharmacol. (2019) 17:33–58. doi: 10.2174/1570159X16666180917105033 PMC6341499 30221603

[17] Ren H Zhao D Xu X Yang Y Fan S Li W . Paraneoplastic cerebellar degeneration associated with anti-protein kinase Cgamma antibodies in a Chinese patient. J Neuroimmunol. (2020) 350:577408. doi: 10.1016/j.jneuroim.2020.577408 33217719

[18] Spatola M Petit Pedrol M Maudes E Simabukuro M Muñiz-Castrillo S Pinto A-L . Clinical features, prognostic factors, and antibody effects in anti-mGluR1 encephalitis. Neurology. (2020) 95:e3012–25. doi: 10.1212/wnl.0000000000010854 32928978 PMC7734921

[19] Titulaer MJ McCracken L Gabilondo I Armangué T Glaser C Iizuka T . Treatment and prognostic factors for long-term outcome in patients with anti-NMDA receptor encephalitis: an observational cohort study. Lancet Neurol. (2013) 12:157–65. doi: 10.1016/s1474-4422(12)70310-1 23290630 PMC3563251

[20] Wu Q Gong B Jiang A Qin X . Case report and literature analysis: Autoimmune cerebellar ataxia associated with homer-3 antibodies. Front Neurol. (2022) 13. doi: 10.3389/fneur.2022.951659 35959384 PMC9360609

[21] Zian Z Berry S-G Bahmaie N Ghotbi D Kashif A Madkaikar M . The clinical efficacy of Rituximab administration in autoimmunity disorders, primary immunodeficiency diseases and malignancies. Int Immunopharmacol. (2021) 95:107565. doi: 10.1016/j.intimp.2021.107565 33773205

[22] Zis P Rao DG Hoggard N Sarrigiannis PG Hadjivassiliou M . Anti-MAG associated cerebellar ataxia and response to rituximab. J Neurol. (2018) 265:115–8. doi: 10.1007/s00415-017-8675-9 29159464

