# Supplementary material for: Association between rituximab treatment and outcome in nonparaneoplastic autoimmune cerebellar ataxia: an observational cohort study
Source: Front Immunol. 2026 Oct 8;17:1931030. doi: 10.3389/fimmu.2026.1931030 (PMC13687354; doi:10.3389/fimmu.2026.1931030)
Supplement: Supplementary file 1 [file Supplementaryfile1.docx]

**Supplementary Table 1 Distribution of anti-neural antibodies**

| ACA subtype | Neural antibodies | No | ACA subtype | Neural antibodies | No |
| --- | --- | --- | --- | --- | --- |
| mGLuR1-CA | Anti-mGluR1 | 12 | PACA |  | 105 |
| GAD-CA | Anti-GAD | 10 |  | TBA positive | 18 |
| AIE-CA |  | 13 |  | Anti-Homer3 | 8 |
|  | Anti-NMDAR | 10 |  | Anti-GFAP | 9 |
|  | Anti-DPPX | 1 |  | Anti-Sez6L2 | 2 |
|  | Anti-CASPR2 | 1 |  | Anti-Yo* | 2 |
|  | Anti-IgLON5 | 1 |  | Anti-CARPVIII | 1 |
| OMS |  | 4 |  | Anti-AP3B2 | 1 |
|  | TBA positive | 1 |  | Anti-Zic4 | 1 |
|  | Antibody negative | 3 |  | Anti-Rab6 | 1 |
|  |  |  |  | Anti-EEF1D | 1 |
|  |  |  |  | Anti-ARHGAP26 | 1 |
|  |  |  |  | Antibody negative | 60 |

* Two patients had low-titer anti-Yo positivity. Comprehensive malignancy screening was negative, with no malignancy identified during at least 3 years of follow-up, and both patients showed clinical improvement after immunotherapy. Based on overall clinical adjudication, they were classified as PACA rather than PCD. CDR2L-specific antibody testing was not completed.

ACA, autoimmune cerebellar ataxia; AIE-CA, autoimmune encephalitis related cerebellar ataxia; GAD-CA, anti-glutamic acid decarboxylase antibody related cerebellar ataxia; mGLuR1-CA, anti-mGluR1 related cerebellar ataxia; OMS, opsoclonus-myoclonus syndrome; PACA, primary autoimmune cerebellar ataxia; TBA, tissue-based assay

**Supplementary Table 2 Revised multivariable linear regression analysis of factors associated with ΔmRS***

| **Variables** | **Regression coefficient, β** | **95% CI** | **P value** |
| --- | --- | --- | --- |
| Age at onset/year | -0.007 | -0.022 to 0.009 | 0.414 |
| Male vs. female | 0.698 | 0.193 to 1.204 | **0.007** |
| Coexisting autoimmune diseases | 0.285 | -0.315 to 0.884 | 0.350 |
| Zenith mRS score | 0.595 | 0.378 to 0.813 | **＜0.001** |
| Immunotherapy |  |  |  |
| RTX | -0.499 | -1.037 to 0.040 | 0.069 |
| Pulse corticosteroid | -0.602 | -1.312 to 0.107 | 0.095 |
| Non-pulse corticosteroid | -0.252 | -0.898 to 0.395 | 0.443 |
| IVIg | -0.039 | -0.652 to 0.573 | 0.899 |
| MMF | -0.364 | -0.853 to 0.124 | 0.142 |
| Follow-up duration/month | -0.012 | -0.021 to -0.003 | **0.011** |

* Effectiveness of first-line immunotherapy and relapse were removed in this revised multivariable linear regression analysis

CI, confidence interval; IVIg, intravenous immunoglobulin; MMF, mycophenolate mofetil; mRS, modified Rankin Scale; RTX, rituximab

R² = 0.269; adjusted R² =0.213

**Supplementary Table 3 Baseline Characteristics Before and After Propensity Score Matching**

| **Variable** | **RTX before** | **Non-RTX before** | **SMD before** | **RTX after** | **Non-RTX after** | **SMD after** |
| --- | --- | --- | --- | --- | --- | --- |
| Male, no. (%) | 21 (42.9) | 38(40.0) | 0.058 | 20 (46.5) | 18 (41.9) | 0.094 |
| Age at onset, years | 35 (23, 45) | 42 (28, 52) | -0.289 | 37 (23, 47) | 40 ( 24, 47) | -0.089 |
| Zenith mRS score | 4 (3.5, 4.5) | 4 (2, 4) | 0.503 | 4 (3, 4) | 4 (3, 4) | -0.070 |

mRS, modified Rankin Scale; RTX, rituximab; SMD, standardized mean difference

**Supplementary Table 4 Multivariable linear regression analysis of factors associated with ΔmRS in the propensity-score-matched cohort**

| **Variables** | **Regression coefficient, β** | **95% CI** | **P value** |
| --- | --- | --- | --- |
| Age at onset/year | -0.003 | -0.026 to 0.021 | 0.827 |
| Male vs. female | 0.462 | -0.241 to 1.164 | 0.194 |
| Coexisting autoimmune diseases | 0.076 | -0.701 to 0.852 | 0.846 |
| Zenith mRS score | 0.413 | 0.062 to 0.764 | **0.022** |
| Immunotherapy |  |  |  |
| RTX | -0.200 | -0.988 to 0.588 | 0.614 |
| Pulse corticosteroid | -0.571 | -1.597 to 0.455 | 0.271 |
| Non-pulse corticosteroid | 0.081 | -0.795 to 0.957 | 0.854 |
| IVIg | 0.591 | -0.318 to 1.499 | 0.199 |
| MMF | -0.266 | -0.959 to 0.428 | 0.448 |
| Effectiveness of first-line immunotherapy | 1.539 | 0.626 to 2.451 | **0.001** |
| Follow-up duration/month | -0.013 | -0.025 to ＜0.001 | **0.046** |
| Relapse | -0.787 | -1.701 to 0.126 | 0.090 |

CI, confidence interval; IVIg, intravenous immunoglobulin; MMF, mycophenolate mofetil; mRS, modified Rankin Scale; RTX, rituximab

R² = 0.376; adjusted R² =0.272

**Supplementary Table 5 Revised Cox regression analysis of predictors of relapse in the time-to-event analysis***

| Variables | HR (95% CI) | P value |
| --- | --- | --- |
| RTX exposure as a time-dependent covariate | 0.657 (0.223 to 1.933) | 0.445 |
| Age at onset/year | 0.960 (0.942 to 0.978) | **<0.001** |
| Male vs. female | 1.075 (0.588 to 1.965) | 0.814 |
| Zenith mRS score | 1.064 (0.805 to 1.405) | 0.664 |

* Effectiveness of first-line immunotherapy was removed in this revised Cox regression analysis

CI, confidence interval; HR, hazard ratio; mRS, modified Rankin Scale; RTX, rituximab

concordance index=0.682

**Supplementary Table 6 Fully adjusted multivariable linear regression analysis of factors associated with ΔmRS in patients with zenith mRS score ≥4**

| **Variables** | **Regression coefficient, β** | **95% CI** | **P value** |
| --- | --- | --- | --- |
| Age at onset/year | -0.004 | -0.023 to 0.015 | 0.650 |
| Male vs. female | 0.976 | 0.405 to 1.547 | **0.001** |
| Coexisting autoimmune diseases | 0.252 | -0.432 to 0.937 | 0.465 |
| Zenith mRS score | 1.368 | 0.388 to 2.347 | **0.007** |
| Immunotherapy |  |  |  |
| RTX | -0.314 | -0.943 to 0.314 | 0.323 |
| Pulse corticosteroid | -0.320 | -1.105 to 0.466 | 0.420 |
| Non-pulse corticosteroid | 0.271 | -0.449 to 0.991 | 0.456 |
| IVIg | -0.109 | -0.862 to 0.644 | 0.774 |
| MMF | -0.607 | -1.196 to -0.018 | **0.043** |
| Effectiveness of first-line immunotherapy | 1.433 | 0.434 to 2.432 | **0.006** |
| Follow-up duration/month | -0.006 | -0.017 to 0.005 | 0.293 |
| Relapse | -1.084 | -1.824 to -0.343 | **0.005** |

CI, confidence interval; IVIg, intravenous immunoglobulin; MMF, mycophenolate mofetil; mRS, modified Rankin Scale; RTX, rituximab

R² = 0.408; adjusted R² =0.313

**Supplementary Table 7 Fully adjusted multivariable linear regression analysis of factors associated with ΔmRS in patients with anti-neural antibodies**

| **Variables** | **Regression coefficient, β** | **95% CI** | **P value** |
| --- | --- | --- | --- |
| Age at onset/year | -0.012 | -0.032 to 0.009 | 0.253 |
| Male vs. female | 0.574 | -0.057 to 1.204 | 0.074 |
| Coexisting autoimmune diseases | 0.297 | -0.533 to 1.128 | 0.478 |
| Zenith mRS score | 0.396 | 0.128 to 0.665 | **0.004** |
| Immunotherapy |  |  |  |
| RTX | 0.658 | -0.050 to 1.365 | 0.068 |
| Pulse corticosteroid | -0.596 | -1.496 to 0.304 | 0.191 |
| Non-pulse corticosteroid | -0.048 | -0.858 to 0.761 | 0.905 |
| IVIg | -0.180 | -0.955 to 0.594 | 0.644 |
| MMF | -0.198 | -0.832 to 0.436 | 0.534 |
| Effectiveness of first-line immunotherapy | 1.075 | 0.257 to 1.894 | **0.011** |
| Follow-up duration/month | -0.019 | -0.030 to -0.007 | **0.002** |
| Relapse | -0.610 | -1.367 to 0.148 | 0.113 |

CI, confidence interval; IVIg, intravenous immunoglobulin; MMF, mycophenolate mofetil; mRS, modified Rankin Scale; RTX, rituximab

R² = 0.393; adjusted R² =0.284

**Supplementary Table 8 Fully adjusted multivariable linear regression analysis of factors associated with ΔmRS in patients without anti-neural antibodies**

| **Variables** | **Regression coefficient, β** | **95% CI** | **P value** |
| --- | --- | --- | --- |
| Age at onset/year | -0.011 | -0.037 to 0.016 | 0.416 |
| Male vs. female | 0.701 | -0.046 to 1.447 | 0.065 |
| Coexisting autoimmune diseases | -0.034 | -0.901 to 0.833 | 0.937 |
| Zenith mRS score | 0.613 | 0.287 to 0.939 | **＜0.001** |
| Immunotherapy |  |  |  |
| RTX | -1.329 | -2.194 to -0.464 | **0.003** |
| Pulse corticosteroid | -0.010 | -1.001 to 0.981 | 0.984 |
| Non-pulse corticosteroid | -0.606 | -1.542 to 0.331 | 0.200 |
| IVIg | -0.359 | -1.254 to 0.536 | 0.424 |
| MMF | 0.043 | -0.686 to 0.772 | 0.906 |
| Effectiveness of first-line immunotherapy | 1.424 | 0.291 to 2.557 | **0.015** |
| Follow-up duration/month | 0.007 | -0.007 to 0.022 | 0.301 |
| Relapse | -1.189 | -2.143 to -0.236 | **0.016** |

CI, confidence interval; IVIg, intravenous immunoglobulin; MMF, mycophenolate mofetil; mRS, modified Rankin Scale; RTX, rituximab

R² = 0.570; adjusted R² =0.467

**Supplementary Table 9 Fully adjusted multivariable linear regression analysis including the RTX × anti-neural antibody status interaction term**

| **Variables** | **Regression coefficient, β** | **95% CI** | **P value** |
| --- | --- | --- | --- |
| Age at onset/year | -0.007 | -0.022 to 0.008 | 0.382 |
| Male vs. female | 0.651 | 0.197 to 1.105 | **0.005** |
| Coexisting autoimmune diseases | 0.148 | -0.414 to 0.710 | 0.604 |
| Zenith mRS score | 0.490 | 0.290 to 0.691 | **<0.001** |
| Immunotherapy |  |  |  |
| RTX | -1.229 | -1.968 to -0.491 | **0.001** |
| Pulse corticosteroid | -0.397 | -1.038 to 0.245 | 0.223 |
| Non-pulse corticosteroid | -0.228 | -0.814 to 0.357 | 0.442 |
| IVIg | -0.098 | -0.655 to 0.459 | 0.728 |
| MMF | -0.230 | -0.684 to 0.225 | 0.319 |
| Effectiveness of first-line immunotherapy | 1.160 | 0.515 to 1.805 | **<0.001** |
| Follow-up duration/month | -0.009 | -0.017 to 0.000 | **0.049** |
| Relapse | -0.778 | -1.338 to -0.218 | **0.007** |
| Anti-neural antibody status (positive vs. negative) | -0.840 | -1.378 to -0.301 | **0.002** |
| RTX × anti-neural antibody status * | 1.797 | 0.874 to 2.719 | **<0.001** |

* Anti-neural antibody status was coded as 1 for antibody-positive patients and 0 for antibody-negative patients. Therefore, the coefficient of RTX represents the association between RTX and ΔmRS among antibody-negative patients, and the RTX × serostatus interaction term represents the difference in the RTX coefficient between antibody-positive and antibody-negative patients.

CI, confidence interval; IVIg, intravenous immunoglobulin; MMF, mycophenolate mofetil; mRS, modified Rankin Scale; RTX, rituximab

R² = 0.436; adjusted R² =0.374

**Supplementary Table 10 Comparison of serum IgG concentration, neutrophile and lymphocyte counts before RTX treatment between RTX responders and non-responders**

|  | **RTX responders (n=30)** | **RTX non-responders (n=13)*** | **P value** |
| --- | --- | --- | --- |
| IgG concentration/g·L^-1^ | 8.34 (7.23, 9.92) | 9.45 (7.84, 10.92) | 0.265 |
| Neutrophile/10^9^·L^-1^ | 5.37 (4.13, 8.38) | 5.56 (3.71, 7.51) | 0.505 |
| Lymphocyte/10^9^·L^-1^ | 1.88 (1.25, 3.03) | 2.64 (1.39. 3.47) | 0.352 |
| CD19/10^6^·L^-1^ | 249 (155, 364) | 425 (195, 657) | 0.116 |
| CD3/10^6^·L^-1^ | 1368 (899, 2261) | 1679 (993, 2527) | 0.744 |
| CD4/10^6^·L^-1^ | 754 (436, 1180) | 953 (608, 1244) | 0.539 |
| CD8/10^6^·L^-1^ | 550 (358, 896) | 552 (304, 1014) | 0.824 |

* Data of serum IgG concentration, neutrophile and lymphocyte counts before RTX treatment were unavailable in two responders and four non-responsers

IgG, immunoglobulin G; RTX, rituximab
